# Prediction of aptamer-protein interacting pairs using an ensemble classifier in combination with various protein sequence attributes

**DOI:** 10.1186/s12859-016-1087-5

**Published:** 2016-05-31

**Authors:** Lina Zhang, Chengjin Zhang, Rui Gao, Runtao Yang, Qing Song

**Affiliations:** School of Control Science and Engineering, Shandong University, Jingshi Road No.17923, Jinan, 250061 China; School of Mechanical, Electrical and Information Engineering, Shandong University at Weihai, Wenhuaxi Road No.180, Weihai, 264209 China; School of Electrical Engineering, University of Jinan, Nanxinzhuangxi Road No.336, Jinan, 250022 China

**Keywords:** Aptamer-protein interacting pairs, Ensemble method, Hybrid features, Imbalanced data problem

## Abstract

**Background:**

Aptamer-protein interacting pairs play a variety of physiological functions and therapeutic potentials in organisms. Rapidly and effectively predicting aptamer-protein interacting pairs is significant to design aptamers binding to certain interested proteins, which will give insight into understanding mechanisms of aptamer-protein interacting pairs and developing aptamer-based therapies.

**Results:**

In this study, an ensemble method is presented to predict aptamer-protein interacting pairs with hybrid features. The features for aptamers are extracted from Pseudo K-tuple Nucleotide Composition (PseKNC) while the features for proteins incorporate Discrete Cosine Transformation (DCT), disorder information, and bi-gram Position Specific Scoring Matrix (PSSM). We investigate predictive capabilities of various feature spaces. The proposed ensemble method obtains the best performance with Youden’s Index of 0.380, using the hybrid feature space of PseKNC, DCT, bi-gram PSSM, and disorder information by 10-fold cross validation. The Relief-Incremental Feature Selection (IFS) method is adopted to obtain the optimal feature set. Based on the optimal feature set, the proposed method achieves a balanced performance with a sensitivity of 0.753 and a specificity of 0.725 on the training dataset, which indicates that this method can solve the imbalanced data problem effectively. To evaluate the prediction performance objectively, an independent testing dataset is used to evaluate the proposed method. Encouragingly, our proposed method performs better than previous study with a sensitivity of 0.738 and a Youden’s Index of 0.451.

**Conclusions:**

These results suggest that the proposed method can be a potential candidate for aptamer-protein interacting pair prediction, which may contribute to finding novel aptamer-protein interacting pairs and understanding the relationship between aptamers and proteins.

**Electronic supplementary material:**

The online version of this article (doi:10.1186/s12859-016-1087-5) contains supplementary material, which is available to authorized users.

## Background

**Fig. 1:**
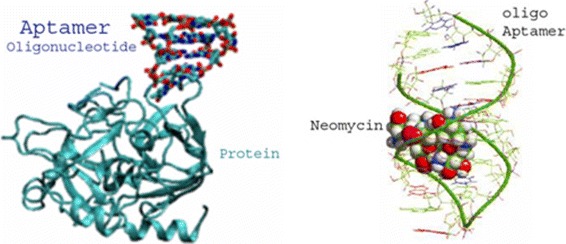
The structures of aptamers binding to specific targets

Aptamers, first reported by Ellington and Gold in 1990 [1, 2], are single stranded DNA/RNA molecules or peptide molecules [3]. They can fold into specific three-dimensional configurations that bind to targets with a high specificity and regulate their activities [4, 5]. The targets include proteins, nucleic acids, drugs, organic dyes, metal ions, and even whole cells or organisms [6, 7]. Figure 1 depicts the structures of two aptamers binding to specific targets. With a deeper understanding of aptamers in terms of their conformational and protein-binding properties, aptamer-protein interacting pairs have a potential to perform a variety of functions [8, 9]. Aptamers exhibit significant advantages over antibodies in flexibility of selection, chemical stability [4], and post-modifications [10].

Since they were discovered, aptamers have garnered tremendous attention and found wide applications in biosensing, target imaging, diagnostics, and therapeutics [11, 12]. In the field of therapy, aptamers are thought to have an excellent potential in treating Age-related Macular Degeneration (AMD) [13], thrombus [14], glomerulonephritis, pulmonary hypertension, and chronic diseases [15, 16]. In the food industry, some aptamers can act as pesticides [17]. What’s more, aptamers have a potential in cancer diagnosis and developing target-based therapeutic drugs delivery to cancer cells, which can reduce side effects of most chemotherapeutic drugs [12].

Due to the physiological functions and practical applications of aptamers, designing aptamers binding to certain interested proteins is crucial to gain insight into mechanisms of aptamer-protein interacting pairs and develop aptamer-based therapies for various diseases. Generally, aptamers can be artificially generated in vitro by a process commonly referred to as SELEX (Systematic Evolution of Ligands by Exponential Enrichment) [18], which consists of several repeated rounds of binding, partition, and amplification [2]. In SELEX experiments, aptamers are identified for their abilities to bind a protein of interest from libraries containing up to 10^16^ different RNA or DNA sequences [19]. Obviously, it is time-consuming and costly to design aptamers for specific proteins using experimental methods. Therefore, it would be of help to develop a computational method for rapidly and effectively predicting the aptamer-protein interacting pairs based on sequence information.

To the best of our knowledge, only one machine learning method has been reported to predict aptamer-protein interacting pairs. Li et al. [20], utilized random forest to establish the prediction model, which integrated information from nucleotide composition, amino acid composition, pseudo-amino acid composition. The maximum Relevance Minimum Redundancy (mRMR) combined with Incremental Feature Selection (IFS) strategy was applied to select high discriminative features. This method has its own merits and does facilitate the development of this field, but achieves severely imbalanced performance with a high specificity and a low sensitivity, which may be attributed to the following shortcomings. (1) Previous study merely extracted composition-based features based on the alphabetic sequences, and failed to capture some sequence-order information. Some useful features based on structural and evolutionary information are also missing. It has been a major focus in bioinformatics to integrate heterogeneous features (in some cases coupled with feature selection to remove redundant and irrelevant features from the original feature sets). For example, Li et al. [21] integrated various sources of features including protein functional domains, protein subcellular locations, and protein-protein interaction information to improve the prediction accuracy of kinase-specific phosphorylation sites. In another work, Wang et al. [22] combined heterogeneous features with a two-step feature selection procedure to improve the prediction performance of caspase substrate cleavage sites. In another recent work, Li et al. [23] attained a promising result for glycosylation site prediction by using heterogeneous feature selection. Generally, multiple features can not only preserve enough discriminative information for protein attribute prediction, but also complement each other to enhance the performance and robustness of a predictor [24]. Therefore, the combination of various features from different sources (heterogeneous features) is a good strategy for constructing classifiers [25]. (2) The existing method was based on an individual classifier whose own inherent defects would lead to unsatisfactory prediction performance [26]. In general, an ensemble predictor that integrates diverse learning policies of multiple basic classifiers can outperform its component classifiers [27]. Therefore, the ensemble predictor has been considered as a promising strategy to improve the prediction performance. (3) The previous method did not deal with the serious class imbalance problem, which would lead to a high prediction accuracy for the majority class but a poor prediction accuracy for the minority class [28]. When there is a big difference between the number of positive samples and the number of negative samples, machine learning algorithms will not have sufficient information to learn a function to distinguish the classes due to the inherent learning biases of the imbalanced dataset [29]. Therefore, balanced dataset is needed for avoiding biases in the machine learning [30].

To address the above limitations and further improve the prediction performance, an ensemble method is developed in this paper to predict the aptamer-protein interacting pairs with Pseudo K-tuple Nucleotide Composition (PseKNC), Discrete Cosine Transformation (DCT), disorder information, and bi-gram Position Specific Scoring Matrix (PSSM). In order to reduce the computational complexity and enhance the prediction accuracy, the Relief-IFS method is employed to select high discriminative features. The ensemble random forest classifier is introduced to deal with the imbalanced dataset problem that exists in predicting the aptamer-protein interacting pairs. 10-fold cross validation is carried out to evaluate the performance of the proposed method. Our method achieves promising prediction performance with a balanced sensitivity and a specificity. Further analysis of the optimal features provides insights into the mechanisms of aptamer-protein interacting pairs.

## Methods

### Data collection

In order to evaluate the proposed method and facilitate its comparison with previous studies in predicting aptamer-protein interacting pairs, we use the benchmark dataset constructed recently by Li et al. [20]. The dataset is obtained from Apatmer Base [31]. It is divided into a training dataset and an independent testing dataset. The training dataset consists of 580 positive and 1740 negative samples while the independent testing dataset consists of 145 positive and 435 negative samples. The samples in the independent testing dataset are not in the training dataset. The training dataset and independent testing dataset are given in Additional file 1.

### Feature extraction

An important issue in designing a predictor is how to convert an input sample sequence into a set of numerical features that are fed into a classifier. Appropriate input representations make it easier for the classifier to recognize underlying regularities, which is vital to the success of classifier learning [32]. In general, an individual feature extraction strategy can only represent partial sample’s characteristics, which may limit the prediction performance. Multiple feature extraction strategies can complement each other to enhance the prediction accuracy.

Since each sample in the current dataset consists of an aptamer (DNA or RNA) and a target protein, PseKNC is adopted to formulate the aptamer sequences while hybrid features extracted from DCT, disorder information, and bi-gram PSSM are utilized for encoding target protein sequences.

#### Represent aptamers with pseudo K-tuple nucleotide composition

Suppose a DNA/RNA sequence *D* with *L* nucleic acid residues, i.e.

1 $$ \begin{array}{l} D = {R_{1}},{R_{2}}, \cdots,{R_{i}}, \cdots,{R_{L}},\\ {R_{i}} \in \{ denine \left(A \right), cytosine \left(C \right), guanine \left(G \right),\\ thymine\left(T \right) or uracil\left(U \right)\}, \end{array}  $$

where *R*_*i*_ denotes the *i*th nucleic acid residue along the given sequence.

Nucleic Acid Composition (NAC) is the most simple feature to encode a DNA/RNA sequence. The sequence *D* can be formulated by NAC as the following feature vector:

2 $$ {F_{1}} = \,[f\left(A \right),f\left(C \right),f\left(G \right),{f\left(T \right)or}f(U)],  $$

where $f\left (A \right), f\left (C \right),f\left (G \right),f\left (T \right)orf(U)$ are the normalized occurrence frequencies of the corresponding nucleotides.

In this type of representation, the sequence order information is completely lost which in turn affects the prediction performance. In order to capture local order information and global sequence-order information, Pseudo K-tuple Nucleotide Composition (PseKNC) [33, 34] is introduced here. Recent studies indicate that PseKNC have been successfully applied in identifying recombination spots [35], promoters [36], and nucleosomes [37]. In this paper, K is set as 2 for dinucleotide and 3 for trinucleotide, respectively.

As known, DNA physicochemical properties have been proved to play a significant impact on gene expression regulation [38]. Therefore, physicochemical properties of nucleotides are used to formulate PseKNC for DNA/RNA sequences. Results in [34] have shown that DNA/RNA dinucleotide physical structures, including twist, tilt, roll, shift, slide and rise, contribute to dealing with DNA/RNA sequences. Therefore, these six dinucleotide physical structures are employed to encode the pseudo 2-tuple nucleotide composition. Meanwhile, 12 physicochemical properties of trinucleotides are all included to encode the pseudo 3-tuple nucleotide composition. The values for both the 6 physicochemical properties of dinucleotides and the 12 physicochemical properties of trinucleotides can be referred to [33].

For PseKNC, the sequence-order information of a DNA/RNA sequence can be reflected by a series of correlation factors, defined as

3 $$ \left\{ \begin{array}{l} {\theta_{1}} = \frac{1}{{L - K}}\sum\limits_{i = 1}^{L - K} {{\Theta_{i,i + 1}}} \\ {\theta_{2}} = \frac{1}{{L - K - 1}}\sum\limits_{i = 1}^{L - K - 1} {{\Theta_{i,i + 2}}} \\ {\theta_{3}} = \frac{1}{{L - K - 2}}\sum\limits_{i = 1}^{L - K - 2} {{\Theta_{i,i + 3}}} \\ \cdots \cdots \\ {\theta_{\lambda}} = \frac{1}{{L - K - \lambda + 1}}\sum\limits_{i = 1}^{L - K - \lambda + 1} {{\Theta_{i,i + \lambda }}} \end{array} \right.\lambda = {L_{\min }} - K,  $$

where

4 $$ {\left\{ \begin{array}{l} {\Theta_{i,i + j}} = \frac{1}{N}\sum\limits_{n = 1}^{N} {{{\left[{H_{n}}\left({R_{i}}{R_{i + 1}} \cdots {R_{i + K - 1}}\right) - {H_{n}}\left({R_{i + j}}{R_{i + j + 1}} \cdots {R_{i + j + K - 1}}\right)\right]}^{2}}} \\ i = 1,2, \cdots,L - K + 1;j = 1,2, \cdots,\lambda \end{array} \right.\!\!,}  $$

where *θ*_*λ*_ is the *λ*th tier correlation factor that reflects the sequence order correlation between all the *λ*th most contiguous K-tuple nucleotides along a DNA sequence. *λ* is the highest rank of correlation factor along the DNA/RNA sequence, and ${L_{\min }}$ is the length of the DNA/RNA sequence with minimum length in the training dataset. Here, we set $\lambda = {L_{\min }} - K$. *Θ*_*i*,*i*+*j*_ is the correlation function; *H*_*n*_(*R*_*i*_*R*_*i*+1_⋯*R*_*i*+*K*−1_) denotes the normalized value of the *n*th physicochemical property for K-tuple nucleotide *R*_*i*_*R*_*i*+1_⋯*R*_*i*+*K*−1_ at position *i* and *H*_*n*_(*R*_*i*+*j*_*R*_*i*+*j*+1_⋯*R*_*i*+*j*+*K*−1_) the corresponding value for K-tuple nucleotide *R*_*i*+*j*_*R*_*i*+*j*+1_⋯*R*_*i*+*j*+*K*−1_ at position *i*+*j*. *N* is the total number of physicochemical properties for K-tuple nucleotides. Here, *N* equals to 6 for pseudo 2-tuple nucleotide composition and *N* equals to 12 for pseudo 3-tuple nucleotide composition.

Finally, a DNA/RNA sequence can be represented by a (4^*K*^+*λ*)-dimensional feature vector using the PseKNC,

5 $$ D_{PseKNC}= \left[{d_{1}}\; \cdots \;{d_{{4^{K}}}}\,{d_{{4^{K}} + 1}}\, \cdots \;{d_{{4^{K}} + \lambda }}\right],(\lambda = {L_{\min }} - K),  $$

where

6 $$  {d_{u}} = \left\{ \begin{array}{l} \frac{{f_{u}^{K - tuple}}}{{{\sum\nolimits}_{i = 1}^{{4^{K}}} {f_{i}^{K - tuple}} + w{\sum\nolimits}_{j = 1}^{\lambda} {{\theta_{j}}} }}\quad \left(1 \le u \le {4^{K}}\right)\\ \frac{{w{\theta_{u - {4^{K}}}}}}{{{\sum\nolimits}_{i = 1}^{{4^{K}}} {f_{i}^{K - tuple}} + w{\sum\nolimits}_{j = 1}^{\lambda} {{\theta_{j}}} }}\quad \left({4^{K}} + 1 \le u \le {4^{K}} + \lambda \right) \end{array} \right.,  $$

where $f_{u}^{K - tuple}$ is the normalized occurrence frequency of the *u*th K-tuple nucleotide. *ω* is the weight factor.

#### Represent target proteins with hybrid features

**Discrete cosine transform** A protein sequence occasionally shows periodicity of hydrophobicity and hydrophilicity, which plays a significant role in protein attribute prediction [39]. To achieve this goal, hydrophobicity and hydrophilicity of amino acids along the protein sequence are employed and transformed into a discrete frequency domain. Then, the frequency information reflecting the periodicity, is merged into a set of discrete components which can be used to identify the distribution of the power contained in a protein sequence over the frequencies [40].

Discrete Cosine Transform (DCT), proposed by Ahmed et al. [41], is a real-valued and quasi-orthogonal transformation approach converting numerical values into frequency domain with lower computational complexities. The strong capability of the DCT to compress energy makes the DCT a good candidate for pattern recognition applications [42].

Based on the hydrophobicity or hydrophilicity of amino acids, the DCT of a given protein sequence with a length of *L* is formulated as

7 $$ \begin{aligned} G(k) &= a(k)\sum\limits_{n = 0}^{L - 1} {H\left({p_{n}}\right)\cos \left[\frac{{(2n + 1)k\pi }}{{2L}}\right]},\\k &= 0,1,2,\cdots,L - 1, \end{aligned}  $$

8 $$ a(k) = \left\{ \begin{array}{l} \sqrt {\frac{1}{L}},k = 0\\ \sqrt {\frac{2}{L}},k \ne 0 \end{array} \right.,  $$

where *G*(*k*),*k*=0,1,⋯,*L*−1 represents the spectral characteristic of the sequence. *G*(0) denotes the constant component and the remaining represent the harmonic components of the sequence.

The low-frequency components of DCT, which preserve the global information along with some sequence order information, contain more biological significance than high frequency noisy ones [39]. As the minimum length of protein sequences in the dataset is 52. For the hydrophobicity or hydrophilicity of amino acids, we use 52 low frequency DCT components to represent protein sequences.

**Bi-gram position specific scoring matrix** According to molecular evolution, protein sequences stem from a very finite number of ancestral species, which evolves undergoing changes, insertions, and deletions of single or several residues [43]. With the accumulation over a long period of time, many similarities between original and resultant protein sequences are gradually eliminated, but the corresponding sequences may still share some structure similarities and the same functions [44]. It is indicated that protein sequence evolutionary conservations serve as evidence for structural and functional conservations. Therefore, evolutionary conservations can determine important biological functions and are important in biological sequence analysis [45].

The position-specific score matrix (PSSM), derived from the Position-Specific Iterative Basic Local Alignment Search Tool (PSI-BLAST) [46], is adopted to obtain the evolutionary conservations. For a given protein sequence with a length of *L*, the corresponding PSSM profile is composed of *L*∗20 elements defined as:

9 $$ {{\rm{P}}_{{\rm{PSSM}}}} = \left[ {\begin{array}{*{20}{c}} {{E_{1 \to 1}}}&{{E_{1 \to 2}}}& \cdots &{{E_{1 \to j}}}& \cdots &{{E_{1 \to 20}}}\\ {{E_{2 \to 1}}}&{{E_{2 \to 2}}}& \cdots &{{E_{2 \to j}}}& \cdots &{{E_{2 \to 20}}}\\ \vdots & \vdots & \cdots & \vdots & \cdots & \vdots \\ {{E_{i \to 1}}}&{{E_{i \to 2}}}& \cdots &{{E_{i \to j}}}& \cdots &{{E_{i \to 20}}}\\ \vdots & \vdots & \cdots & \vdots & \cdots & \vdots \\ {{E_{L \to 1}}}&{{E_{L \to 2}}}& \cdots &{{E_{L \to j}}}& \cdots &{{E_{L \to 20}}} \end{array}} \right],  $$

where the rows and columns of the matrix are indexed by the protein residues and the 20 native amino acids, respectively. The values in the *i*th row denote the probabilities of the *i*th residue in the given protein sequence mutating to the 20 native amino acids during the evolution process. PSSM generally contains positive or negative integers. Positive scores indicate that the given amino acid substitution occurs more frequently than expected occasionally while negative scores indicate the opposite [47]. What’s more, large positive scores often represent active sites required for other intermolecular interactions [48].

We extract bi-gram features from PSSM [49] to represent protein sequences, which are defined as

10 $$ {B_{m,n}} = \sum\limits_{i = 1}^{L - 1} {{P_{i \to m}}{P_{i + 1 \to n}}},(m,n = 1,2, \cdots,20),  $$

where *B*_*m*,*n*_ denotes the frequency of transition from the *m*th amino acid on the *i*th position to the *n*th amino acid on the (*i*+1)th position. These features incorporate neighborhood information of amino acids and evolutionary information from PSSM.

Eventually, 400 frequencies can be obtained and formulated as

11 $$ \begin{aligned} {F_{PSSM}} =&\, [{B_{1,1}}, \cdots,{B_{1,20}},{B_{2,1}}, \cdots,{B_{2,20}}, \cdots \cdots,\\ &{B_{20,1}}, \cdots,{B_{20,20}}]. \end{aligned}  $$

**Disorder information** Protein segments are defined as unstructured or disordered if they lack stable three-dimensional structures or if they have a large number of conformations under physiological conditions [50]. Such disordered regions of proteins allow for more modification sites and flexible interaction partners. Therefore, the information of disorder regions is of great importance for the functions and structure forming of proteins [51]. In this study, VSL2 [52], which is one of the best disorder predictor and can accurately predict both long and short disordered regions in proteins, is employed to calculate the disorder score for each residue. Disorder score reflects the disorder status of each amino acid in a given protein sequence. The disorder score ranges from 0 to 1. The higher score represents the corresponding residue is more likely to lack fixed structure.

The length of disorder scores for each protein sequence is varying, which is inappropriate to develop a predictor. Auto covariance (AC), depicting the average interactions between two residues, has been successfully adopted to grasp the local discriminative information [53]. To solve the variable dimension problem, AC descriptors are adopted here to acquire more local sequence order information.

For a protein sequence with the length of *L*, disorder scores are obtained with the same length from VSL2, defined as

12 $$ [{d_{1}}, \cdots,{d_{i}}, \cdots,{d_{L}}],  $$

where *d*_*i*_ denotes the disorder score of the residue on the *i*th position along the given protein sequence.

To extract features from the disorder score, AC is defined as

13 $$ \begin{aligned} A{C_{\lambda}} =&\, \frac{1}{{(L - \lambda)}}\sum\limits_{i = 1}^{L - \lambda} {({d_{i}} - \overline d)*({d_{(i + \lambda)}} - \overline d)},\\&(\lambda = 1,2,...,{L_{\min }} - 1), \end{aligned}  $$

where $\bar {d}$ is the average value of the disorder score vector; *λ* is the distance between two considered amino acid residues, which is closely related to sequence order information and plays an important role in the performance of a predictor. *L*_*min*_ is the length of the protein sequence with the minimum length which equals to 52 in this study. From the above equation, 51 order-based features are calculated. To extract more disorder-based feature, the following features can be obtained. (i) mean/standard deviation of all residues disorder scores (2 features); (ii) number of disorder/non-disorder segments (2 features); (iii) minimum/maximum length of disorder/non-disorder segments (4 features). Therefore, 59 disorder-based features can be obtained to represent proteins.

### Feature selection

After carrying out the above feature extraction methods, all the aptamer-protein interacting pairs with various lengths are converted into numerical feature vectors with the same dimension. However, not all the extracted features can contribute equally to classification. There may have some uncorrelated and redundant information among the extracted features, which can affect the speed and prediction performance of a predictor [54]. Feature selection techniques are essential to pick out informative features and gain deeper insights into intrinsic properties of protein sequences, which can prevent overfitting, improve the prediction quality, and build a robust prediction model [55]. In this study, the Relief algorithm combined with Incremental Features Selection(IFS) is employed to acquire more discriminative features for predicting aptamer-protein interacting pairs.

**Relief** The Relief algorithm, originally proposed by Kira [56], is considered one of the most successful algorithms for depicting the relevance between the features and class labels. It is noise-tolerant and requires only linear time. The Relief algorithm can be used to estimate feature weights according to the ability of the feature to distinguish the near samples [57]. The Relief algorithm is executed iteratively. During each iteration process, the Relief algorithm endows each feature with a weight as formulated by

14 $$ W_{p}^{i + 1} = {W_{p}^{i}} - \frac{{diff(Y,{x_{i}},H({x_{i}}))}}{m} + \frac{{diff(S,{x_{i}},M({x_{i}}))}}{m},  $$

15 $$ diff(*,x,y) = \left\{ \begin{array}{ll} \left\| {x - y} \right\|, & x \ne y\\ 0, & x = y \end{array} \right.,  $$

where ${W_{p}^{i}}$, $W_{p}^{i + 1}$ denote the current and next weight values, respectively; *p* represents a given feature; *x*_*i*_ stands for the *i*th sample; *H*(*x*_*i*_) represents the nearest neighbor samples from the same class label against *x*_*i*_ (termed the nearest hit); *M*(*x*_*i*_) stands for the nearest neighbor samples from different class labels against *x*_*i*_, (termed the nearest miss). *Y* and *S* denote the sample sets with the same and different class labels against *x*_*i*_, respectively; *m* is the number of random samples; The function of *diff*(∗,*x*,*y*) is used for calculating the distance between the random samples to find the nearest neighbor one.

Relief endows each feature a weight value within range [0, 1]. The feature with a larger weight value indicates that it is a more highly relevant one for the target to be predicted. In other words, predicted targets have a stronger correlation with the *j*th feature than that with the *i*th feature if ${W_{{f_{j}}}} > {W_{{f_{i}}}}$.

The ranked feature list can be obtained based on feature weight values, represented as

16 $$ F = \{ {f_{1}},{f_{2}}, \cdots,{f_{N}}\},  $$

where *f*_1_ is the feature with the highest value of *W*, *f*_2_ with the second highest value of *W*,…, *f*_*N*_ with the lowest value of *W*.

The WEKA (Waikato Environment for Knowledge Analysis) software package [58] is used for the feature selection algorithm of relief, where default parameters are employed.

**Incremental feature selection** Base on the ranked feature list according to the relevance to the class evaluated by the relief algorithm, the incremental feature selection (IFS), one of the well-known searching strategies of feature selection, is employed to determine the optimal features [59]. The IFS procedure starts with an empty subset, and adds features one by one from higher to lower rank into the feature subset. A new feature subset is generated when another feature has been added. The *i*th feature subset can be formulated as

17 $$ {F_{i}} = \{ {f_{1}},{f_{2}}, \cdots,{f_{i}}\} (1 \le i \le N).  $$

For each feature subset *F*_*i*_, an ensemble predictor is constructed and evaluated using 10-fold cross validation test. The IFS curve can be drawn with Youden’s index values as the *y*-axis and index *i* of *F*_*i*_ as the *x*-axis. The feature subset that yields the best prediction performance is determined as the final input of the classification system.

### Ensemble learning method

As illustrated in ’Data collection’, the data imbalance problem exists in predicting aptamer-protein interacting pairs. Previous research has shown that imbalanced datasets are problematic when constructing classifiers [60], which would result in a high prediction accuracy for the majority class but a poor prediction accuracy for the minority class [61, 62]. For example, the predictor in [20] yields serious imbalance performance, with a high specificity of 0.922, but a very low sensitivity of 0.488, and even a relatively high accuracy of 0.813. Many researches [63, 64] extract a very small fraction of the negative samples randomly as the training data, which has changed the distribution of positive and negative samples. This method can’t take full advantage of the most information in the original data, which will lead to a biased estimate of the accuracy. Therefore, the ensemble learning method is used to resolve the imbalanced problem.

**Fig. 2:**
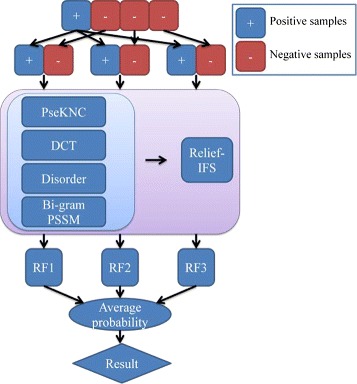
The whole procedure of the ensemble RF classifier. PseKNC: Pseudo K-tuple Nucleotide Composition; DCT: Discrete Cosine Transform; Bi-gram PSSM: Bi-gram Position Specific Scoring Matrix; IFS: Incremental Feature Selection; RF: Random Forest

An ensemble classifier is a collection of multiple basic individual classifiers with diverse learning policies, which is supposed to significantly improve the performance of a prediction method due to the fact that ensemble classifier is able to make use of the different decision boundaries generated from the individual classifiers to strategically combine the classification results [65]. Hansen [66] has demonstrated why an ensemble method gives a much better performance than its component individual classifiers in theory. In order to improve the prediction performance and deal with the data imbalance problem, we employ an ensemble classifier to predict aptamer-protein interacting pairs. The negative samples are divided into *N* parts, and *N* is determined by

18 $$ N={N}_{negative}/{N}_{positive}, $$

where *N*_*negative*_ and *N*_*positive*_ are the numbers of negative and positive samples, respectively. Here, *N* equals to 3. Then, each negative part with the same number of positive samples is combined with the positive samples to construct a sub-training dataset. Three RF models are trained by the 3 sub-training datasets, respectively. The ultimate prediction result of the ensemble RF classifier is determined by the average probability of the outputs of the 3 RF models. This method takes advantage of the information available in the non-aptamer-protein interacting pairs as much as possible to construct the prediction model, so the prediction result is more objective. The whole procedure of the construction of the ensemble RF classifier is shown in Fig. 2.

### Performance measures

In the statistical prediction, there are 3 cross-validation methods often used for examining the accuracy, including independent dataset test, sub-sampling test (e.g. 5-fold or 10-fold cross validation), and jackknife test [67]. Among these three methods, the jackknife test is deemed the most objective and rigorous one that can exclude the memory effects during the entire testing process and can always yield a unique result for a given benchmark dataset, as elucidated in [68] and demonstrated by Eq. 50 of Chou and Shen [69]. Therefore, the jackknife test has been increasingly and widely adopted by investigators to test the power of various prediction methods [70, 71]. To reduce the computational complexity and compare with the existing method objectively, 10-fold cross validation is implemented in this study.

During the procedure, the training dataset is randomly separated into 10 equally-sized parts. Each time, one part is for testing and the other nine parts form the training dataset. This process is repeated ten times to test each part. The ultimate result is the average of the 10 prediction results. To assess the performance of the predictor intuitively, sensitivity (*Sn*), specificity (*Sp*), accuracy (*Acc*), and Matthew’s Correlation Coefficient (*MCC*) are employed, which are defined as

19 $$ Sn = \frac{{TP}}{{FN + TP}},  $$

20 $$ Sp = \frac{{TN}}{{FP + TN}},  $$

21 $$ Acc = \frac{{TN + TP}}{{TN + FP + FN + TP}},  $$

22 $$  MCC = \frac{{TP*TN - FP*FN}}{{\sqrt {(TP + FN)(TP + FP)(TN + FP)(TN + FN)} }},  $$

where *TP*, *FP*, *TN* and *FN* represent true positive (correctly predicted aptamer-protein interacting pairs.), false positive (non aptamer-protein interacting pairs incorrectly predicted as aptamer-protein interacting pairs), true negative (correctly predicted non aptamer-protein interacting pairs), and false negative (aptamer-protein interacting pairs incorrectly predicted as non aptamer-protein interacting pairs), respectively.

Additionally, due to the distinct numbers of positive samples and negative samples in the training dataset, Youden’s Index [72] is used for gaining insights into the relative performance of predictors in general, defined as

23 $$ J = Sn + Sp - 1.  $$

Youden’s index gives the probability of an informed decision and is advantageous as it offers comparison of aptamer-protein interacting pair prediction quality by means of a single informative parameter [73].

## Results and discussions

### Performance analysis of ensemble learning method using different feature spaces

In order to explore the effectiveness of various feature spaces, the prediction results obtained by hybrid feature spaces using 10-fold cross validation are listed in Table 1. The feature space of DCT and PseKNC identifies aptamer-protein interacting pairs with a sensitivity of 0.621 and a Youden’s index of 0.261. DCT, incorporating global information along with some sequence order information, results in an acceptable discrimination power. The feature space of PseKNC and bi-gram PSSM discriminates aptamer-protein interacting pairs with the best performance among the first 3 feature spaces with a sensitivity of 0.660 and a Youden’s index of 0.333. Bi-gram PSSM, considering evolution and order information of the protein sequences, yields a satisfactory prediction performance. The bi-gram PSSM information also has shortcomings. The generation of PSSM of a protein depends largely on the searching dataset. If no homologous sequence is found in the searching dataset, the PSSM can not be obtained [74]. In the implementation process of our proposed method, when there is no homologous of a given protein in search dataset, we assign a zero matrix to the PSSM of the protein. As a minority of sequences have no homologous sequences in the benchmark dataset, the overall prediction performance of the ensemble method will not be affected. In Table 1, the discrimination power of disorder is weaker compared to that of the other two feature spaces, due to the fact that the sequence order information based on disorder along the sequence may not have enough information for identifying aptamer-protein interacting pairs.

**Table 1:** Prediction performance of the ensemble RF models using various feature spaces by 10-fold cross validation

Features	*Sn*	*Sp*	*Acc*	*MCC*	Youden’s index
PseKNC+DCT	0.621	0.641	0.636	0.229	0.261
PseKNC+Bi-gram PSSM	0.660	0.673	0.670	0.293	0.333
PseKNC+Disorder	0.103	0.321	0.267	-0.499	-0.575
PseKNC+DCT+Bi-gram PSSM	0.693	0.666	0.673	0.315	0.359
PseKNC+DCT +Disorder	0.597	0.616	0.611	0.186	0.213
PseKNC+Bi-gram PSSM+Disorder	0.671	0.675	0.674	0.304	0.345
PseKNC+DCT+Bi-gram PSSM+Disorder	0.7	0.680	0.685	0.334	0.380

As shown in Table 1, the hybrid feature space of PseKNC, DCT and bi-gram PSSM achieves a better prediction performance compared to that of PseKNC+DCT and that of PseKNC+bi-gram PSSM. The same result occurs in the hybrid feature space of PseKNC, bi-gram PSSM and disorder. However, the hybrid feature space of PseKNC, DCT and disorder obtains a sensitivity of 0.597 and a Youden’s index of 0.213, worse than those of PseKNC+DCT, but better than those of PseKNC+disorder. This phenomenon may be due to that disorder introduces some redundancy features in the hybrid feature space of PseKNC, DCT and disorder. Furthermore, the hybrid feature space of PseKNC incorporating DCT, bi-gram PSSM and disorder yields the highest sensitivity of 0.7 and the highest Youden’s index of 0.380, indicating the powerful discriminant ability of the ensemble method using the hybrid feature space. Other measures also show the case. These results reveal that different feature spaces extract diverse types of information from different sources and contribute to the prediction accuracy differently. Any feature spaces that may show a poor performance on certain protein attribute prediction cannot be declared as non-discriminative features. They may contain some important information that might be missed by other powerful feature extraction techniques. The hybrid feature spaces can complement each other to enhance the prediction performance of a predictor. Therefore, this study uses the hybrid feature space of PseKNC combining DCT, bi-gram PSSM and disorder to construct the prediction model.

### Solving imbalanced dataset problem

Based on the results of individual RF modules, the ensemble RF classifier attempts to combine different models into a consensus classifier by the average probability of the outputs of the 3 RF models. To evaluate the effectiveness of our ensemble method to overcome the imbalanced problem, Table 2 shows the prediction results with or without the ensemble method by means of the hybrid feature space of PseKNC combining DCT, bi-gram PSSM and disorder.

**Table 2:** Prediction results with or without the ensemble method

Method	*Sn*	*Sp*	*Acc*	*MCC*	Youden’s index
With ensemble	0.7	0.680	0.685	0.334	0.380
Without ensemble	0.3	0.993	0.819	0.465	0.293

As shown in Table 2, without the ensemble method, the accuracy and specificity achieve as high as 0.819 and 0.993, respectively. But the sensitivity and Youden’s index are only 0.3 and 0.293, respectively. The ensemble method achieves a more balanced sensitivity of 0.7 and specificity of 0.680. The value of Youden’s index is 0.380, better than that without ensemble method. The accuracy and *MCC* obtained with the ensemble method are lower than those without ensemble method, which may be due to the imbalanced data size. For the classification of imbalanced data, accuracy and *MCC* are both not appropriate measures because they may be still high when the sensitivity is very low. These results suggest that the ensemble method can solve the imbalanced problem effectively.

### Feature selection results

**Fig. 3:**
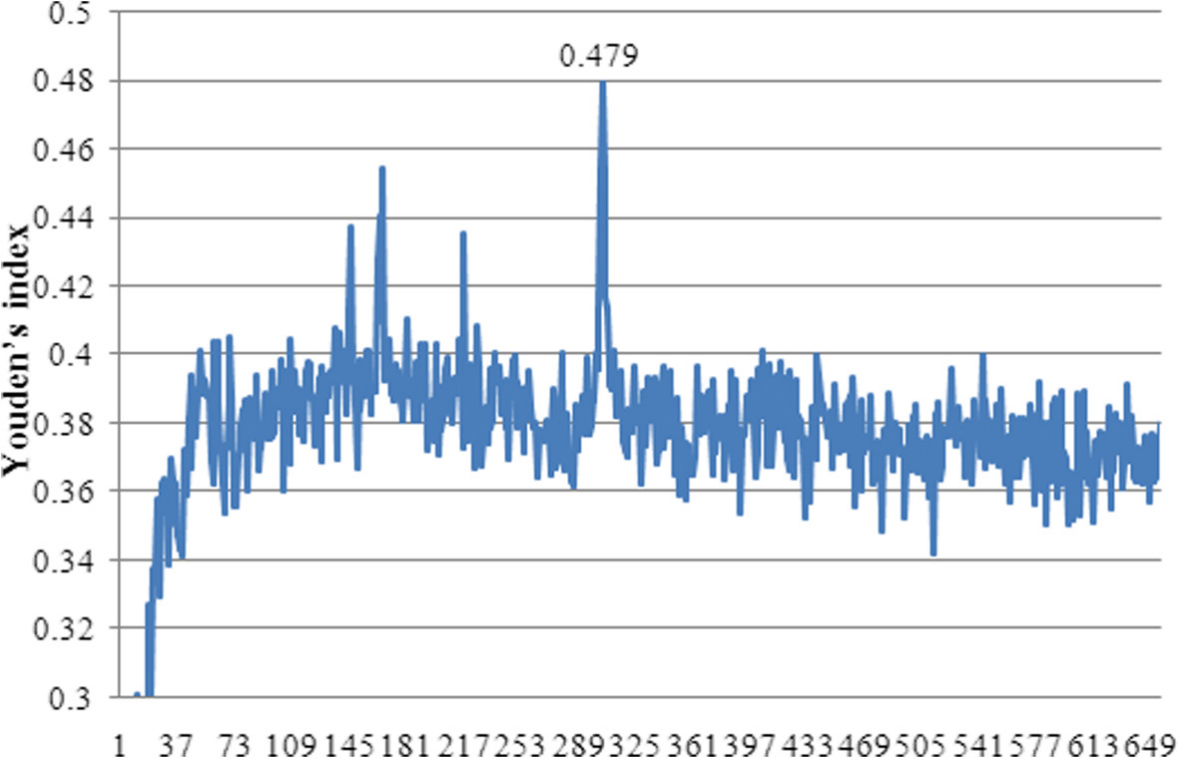
The IFS(Incremental Feature Selection) curve. The values of Youden’s index against the number of features

The ranked feature list of the hybrid feature space of PseKNC combining DCT, bi-gram PSSM and disorder is obtained according to their relevance to the classes based on the Relief method. Within the list (see Additional file 2), a feature with a smaller index represents a more important one for aptamer-protein interacting pair prediction. The feature list is utilized to select the optimal feature subset in the following IFS procedure. Based on the ranked feature list, adding the ranked features one by one, individual predictors for different feature subsets are constructed using the ensemble FR classifier and evaluated by 10-fold cross validation. The IFS results are given in Additional file 3. Then, the IFS curve is plotted in Fig. 3, which shows the relationships of feature indices against Youden’s index. The curve reaches its peak at 0.479 when the top 304 features in Additional file 2 are selected. Thus, these 304 features are regarded as the optimal features for the ensemble RF classifier.

To investigate the influence of feature selection on the performance of the ensemble RF classifier, the prediction performance of the ensemble method with and without feature selection based on hybrid feature space of PseKNC combining DCT, bi-gram PSSM and disorder is shown in Table 3. As can be seen from Table 3, the ensemble method with feature selection achieves a sensitivity of 0.753, a specificity of 0.725, an accuracy of 0.732, a *MCC* of 0.424, and a Youden’s index of 0.479 based on the 304 features, which are all superior to those of the ensemble method without feature selection. These results demonstrate that the original feature set really contains redundant information or noise. The Relief-IFS method can significantly remove these useless features to greatly improve the performance of the ensemble model. The ensemble learning method with feature selection is determined as the final predictor for aptamer-protein interacting pair prediction.

**Table 3:** Performance of the ensemble FR classifier with and without feature selection

Method	No. of features	*Sn*	*Sp*	*Acc*	*MCC*	Youden’s index
Without feature selection	654	0.7	0.680	0.685	0.334	0.380
With feature selection	304	0.753	0.725	0.732	0.424	0.479

### Analysis of the optimal features

**Fig. 4:**
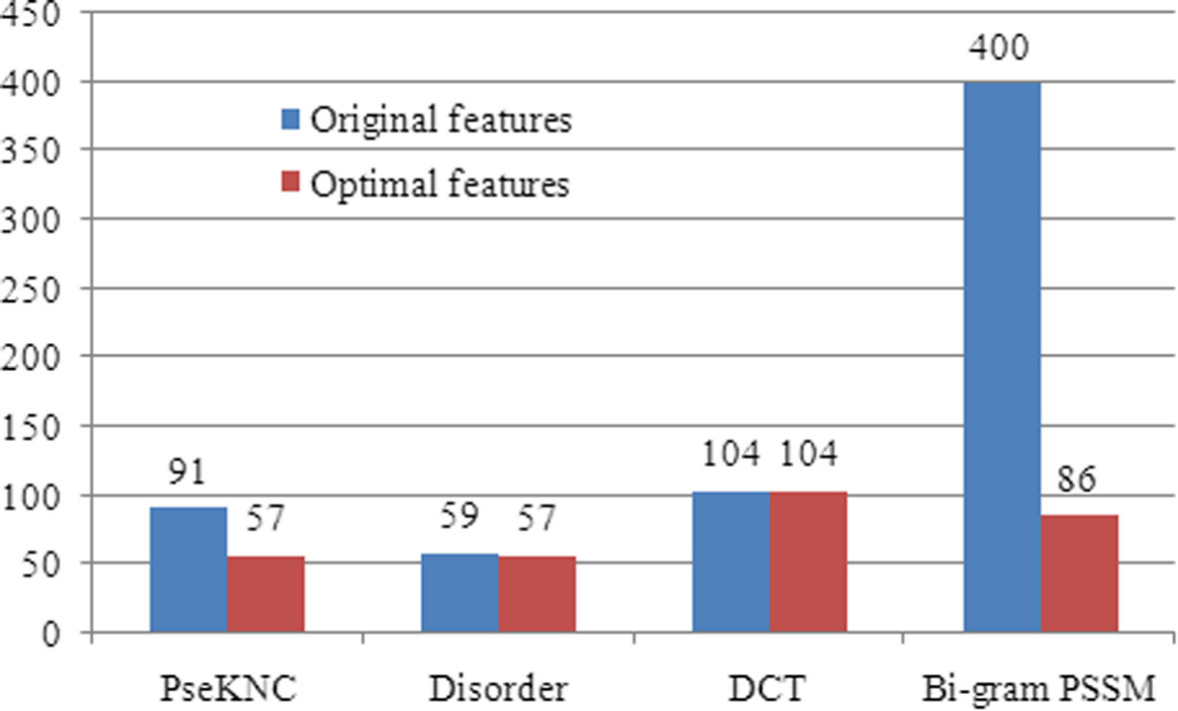
The feature type distribution of the original features and the optimal features

The feature type distributions of the original features and the optimal features are investigated and shown in Fig. 4. There are 57 PseKNC features, 57 disorder features, 104 DCT features, and 86 bi-gram PSSM features in the optimal feature set, indicating that all kinds of features contribute to the prediction of aptamer-protein interacting pairs. The percentages of the optimal features accounting for the corresponding feature types are also investigated, which are 0.626 for PseKNC, 0.966 for disorder, 1.00 for DCT and 0.215 for bi-gram PSSM. It is interesting to note that all DCT features are in the optimal feature set, indicating that DCT based features play a crucial role in predicting aptamer-protein interacting pairs. This is the first attempt to employ DCT based features for aptamer-protein interacting pair prediction, which may help provide new annotations for the properties of these interaction pairs. An overwhelming majority of disorder features (0.966) are selected as the optimal features. It is suggested that disorder based features act an irreplaceable role in the prediction of aptamer-protein interacting pairs. These results indicate that disordered regions of a protein are closely related with the formation of the interaction of an aptamer and a protein, which is in accordance with the statement that disorder information of proteins are of great importance for the functions and structure forming. More than half of features are selected from PseKNC (0.626). This implies that the nucleotide composition and order information play some roles in predicting aptamer-protein interacting pairs. It is noted that a minority of bi-gram PSSM features (0.215) are selected from the original bi-gram PSSM features, due to the fact that the number of this feature type in the original feature set is the most of all those of other feature types. Results in ’Performance analysis of ensemble learning method using different feature spaces’ indicate that they perform a nonnegligible role in improving the prediction performance of the ensemble method.

### Comparison with existing method

The existing method for identifying aptamer-protein interacting pairs [20] present prediction results by using the same size dataset (580 positive and 1740 negative samples) and same validation method (10-fold cross validation). To evaluate the prediction performance objectively, we compare our method with reference [20] on the training dataset. The performance comparison based on the same dataset is much more reliable, which can reflect the performance of a predictor more objectively. Table 4 reports the detailed prediction results obtained by the aforementioned 2 methods. As we can see from Table 4, [20] obtains an imbalanced performance with a low sensitivity of 0.488, but a high specificity of 0.922, indicating that positive samples tend to be identified incorrectly as the negative ones. This study achieves a balanced performance, with a sensitivity of 0.753 and a specificity of 0.725. The sensitivity of this study is far better than that of reference [20]. It is noted that due to the large number of negative samples, the negative samples tend to be identified correctly, which will lead to a large Acc value and a large *MCC* value as given in [20]. As mentioned above, *Acc* and *MCC* are not proper and objective indexes for this serious data imbalance problem. In addition, Youden’s index of this study is better than that of [20]. Overall, the proposed ensemble method achieves a satisfactory performance and can play a complementary role to identify aptamer-protein interacting pairs.

**Table 4:** Performance comparison with the existing method on the training dataset by 10-fold cross validation

Method	*Sn*	*Sp*	*Acc*	*MCC*	Youden’s index
[20]	0.488	0.922	0.813	0.461	0.410
This study	0.753	0.725	0.732	0.424	0.479

To further assess the prediction performance of the proposed method, it is essential to compare the performance of the present method with that of the previous predictor on the same independent testing dataset. The prediction results are summarized in Table 5. In Table 5, though the specificity (0.871) yielded by [20] is better than that (0.713) obtained by our method, the sensitivity (0.483) of [20] is far worse than that (0.738) of our method, which indicates that the imbalance between sensitivity and specificity exists in [20]. Our method achieves a balanced performance with sensitivity of 0.738 and specificity of 0.713, which is also proved by the Youden’s index of 0.451. It is worth pointing out that the proposed ensemble method has a fairly good prediction performance and prediction robust in predicting aptamer-protein interacting pairs.

**Table 5:** Performance comparison with the existing method on the independent testing dataset

Method	*Sn*	*Sp*	*Acc*	*MCC*	Youden’s index
[20]	0.483	0.871	0.774	0.372	0.354
This study	0.738	0.713	0.719	0.398	0.451

### Case study

In the case study section, we select two aptamer-protein interacting pairs identified correctly by our proposed method and analyze their physiological functions. For example, 17155909-human interleukin-23-2 interacting with human-interleukin-23 [75], an aptamer-protein interacting pair, can perform functions in congenital immunity and make a response to infection in organisms. It may not only be responsible for autoimmune inflammatory diseases but also be important for tumorigenesis [75]. Another aptamer-protein interacting pair, 20387790-PAI-1-2 interacting with plasminogen-activator-inhibitor-1 [76], may function as a major control point in the regulation of fibrinolysis and blood coagulation system, regarded as a key marker for cardiovascular diseases [77]. Our proposed method can effectively identify aptamer-protein interacting pairs annotated and reviewed using experimental methods, which is of great theoretical significance in guiding research on aptamer-protein interacting pairs and relevant therapy.

## Conclusions

In this paper, an ensemble method has been presented with a combination of sequence descriptors extracted from PseKNC, DCT, disorder information, and bi-gram PSSM to predict the aptamer-protein interacting pairs. To solve the dimension disaster and improve the prediction capability of the model, the Relief-IFS method is adopted to obtain the optimal feature set. By investigating predictive capabilities of various feature spaces, the proposed ensemble method obtains the best sensitivity of 0.7, specificity of 0.680, and Youden’s Index of 0.380, with the hybrid feature space of PseKNC, DCT, bi-gram PSSM, and disorder information by 10-fold cross validation. These results indicate that the hybrid feature space can complement each other to enhance the prediction performance and the ensemble method can solve the imbalanced problem effectively. The Relief-IFS method can significantly remove useless features to greatly improve the performance of the ensemble model. Analysis of optimal features reveals that all feature types play roles in the determination of aptamer-protein interacting pairs, which may help understand the mechanism of aptamer-protein interactions and provide guidelines for experimental validation. To evaluate the prediction performance objectively, the proposed method is compared with previous study on the same training dataset and independent testing dataset, respectively. Our method obtains a balanced performance. The sensitivity yielded by our method is far better than that achieved by the previous method. In addition, Youden’s index of this study is better than that of the existing method. It is convinced that the proposed method is an effective and powerful approach for predicting aptamer-protein interacting pairs. Since user-friendly and publicly accessible webservers represent the future direction for developing more practically predictors, we will attempt to provide a webserver in our future work for the method presented in this paper.

## Abbreviations

AMD, age-related macular degeneration; Bi-gram PSSM, bi-gram position specific scoring matrix; DCT, discrete cosine transform; IFS, incremental feature selection; mRMR, maximum relevance minimum redundancy; MCC, Matthew’s correlation coefficient; NAC, nucleic acid composition; PseKNC, pseudo K-tuple nucleotide composition; PSI-BLAST, position-specific iterative basic local alignment search tool; RF, random forest; SELEX, systematic evolution of ligands by exponential enrichment

## Additional files

Additional file 1 The benchmark dataset. The training dataset contains 580 positive and 1740 negative samples while the independent testing dataset consists of 145 positive and 435 negative samples. (XLS 441 kb)

Additional file 2 The ranked feature list given by the Relief algorithm. Within the list, a feature with a smaller index indicates that it is more important for aptamer-protein interacting pair prediction. Such a list of ranked features are used to establish the optimal feature set in the IFS procedure. (XLS 56.5 kb)

Additional file 3 The Incremental Feature Selection (IFS) result. By adding features one by one from higher to lower rank, 654 different feature subsets are obtained. The ensemble predictor is then accordingly built for each feature subset and evaluated by 10-fold cross validation. (XLS 125 kb)
